# miR-378a-5p targeting BRAF regulates CD4^+^T cells differentiation to Th1 under r*Eg*.P29 induction

**DOI:** 10.3389/fimmu.2025.1620225

**Published:** 2025-09-04

**Authors:** Tingting Zhang, Hu Mu, Chuan Wang, Bingshuo Qian, Mingxing Zhu

**Affiliations:** ^1^ School of Clinical Medicine, Ningxia Medical University, Yinchuan, China; ^2^ Key Laboratory of Common Infectious Disease Prevention and Control in Ningxia, Ningxia Medical University, Yinchuan, China; ^3^ School of Inspection, Ningxia Medical University, Yinchuan, China; ^4^ School of Basic Medical Sciences, Ningxia Medical University, Yinchuan, China; ^5^ General Hospital of Ningxia Medical University, Ningxia Medical University, Yinchuan, China

**Keywords:** *Echinococcus granulosus* P29, miR-378a-5p, BRAF, CD4^+^T cells differentiation, MAPK/ERK pathway

## Abstract

**Introduction:**

Cystic echinococcosis (CE) is a globally distributed zoonotic disease caused by Echinococcus granulosus (Eg) that often presents with insidious onset and asymptomatic progression. Although several Eg-based recombinant vaccines have been developed for the prevention of CE, our previous study demonstrated that recombinant Eg.P29 (r*E*g.P29) is a potent immunogen that induces a robust Th1 immune response. Furthermore, microarray data from miRNA profiling of CD4^+^T cells isolated from mouse spleens showed that miR-378a-5p was significantly upregulated one week after immunization with r*E*g.P29.

**Methods:**

In this context, bioinformatics predictions and dual-luciferase reporter assays identified BRAF as a direct miR-378a-5p target, with downstream signaling involving the MAPK/ERK pathway.

**Results:**

Our research demonstrated that r*E*g.P29 immunization increased miR-378a-5p expression in naïve CD4^+^T cells, reduced BRAF, MEK1/2, and ERK1/2 expression, and promoted Th1 differentiation while inhibiting Th2 differentiation. Overexpression of miR-378a-5p in naïve CD4^+^T cells yielded similar results, whereas knockdown of miR-378a-5p had the opposite effect.

**Conclusion:**

In summary, our findings reveal that under the induction of r*E*g.P29, miR-378a-5p targeted to BRAF regulation and initiated the differentiation of CD4^+^T cells in mouse spleen to Th1 direction, and MAPK/ERK pathway may be involved in this process, identifying miR-378a-5p as apotential biomarker and immunomodulatory target in CE.

## 1 Introduction

Cystic echinococcosis (CE) is a zoonotic parasitic disease caused by the larval forms of *Echinococcus* tapeworms. It is common in livestock-raising regions such as Central Asia, North and Central Africa, and southwestern Latin America (1). In China, it is primarily found in the western regions, including Xinjiang, Qinghai, Gansu, Ningxia, and Tibet (2, 3). This disease leads to significant health and economic losses for both humans and animals (4–6).

The life cycle of *Echinococcus* spp. involves two types of mammals. CE often causes no apparent symptoms in its early stages and can remain undetected in the host for extended periods (7–9). Current clinical diagnoses of cystic CE largely rely on imaging techniques, which hinders early diagnosis, as hydatid cysts in the liver are slow growing (10). Therapeutic strategies for cystic CE primarily involve surgical intervention and pharmacological treatment; however, complete clinical recovery remains difficult due to the high recurrence rate and potential complications associated with both modalities (11–13). Therefore, vaccine development is essential for preventing CE. For example, *Eg*95 provides 96%–98% protection in sheep (14), and r*Eg*.P29 shows 96.6% and 94.5% protective rates in mice and sheep, respectively (15–17). r*Eg*.P29 induces both humoral and cellular immune responses, stimulating high levels of specific antibodies and promoting Th1 differentiation, which produces IFN-γ and other cytokines to eliminate pathogens (18, 19). However, the precise molecular mechanisms underlying r*Eg*.P29-mediated immunomodulation remain unknown. This study aimed to investigate the molecular mechanisms underlying CD4^+^T cells differentiation and the associated signaling pathways involved in this immunomodulatory process.

When infected by *Echinococcus* larvae, the host mounts a sophisticated immunological response. CD4^+^T cells are crucial in the interaction between *Echinococcus* and its host (20–23). In response to pathogen invasion, naïve CD4^+^T cells develop into Th1 and Th2 cells, with Th cell differentiation and activation having a substantial influence on the outcome of the immune response (24–26).

MicroRNAs, single-stranded non-coding RNAs approximately 22 nucleotides long, are widely expressed in cells. They mainly bind to the 3’ untranslated region of target genes to degrade mRNA or inhibit translation, thereby regulating gene expression (27–31). Recent studies have demonstrated that miRNAs are involved in various diseases (32–36), including host responses to parasitic infections, by modulating gene expression to evade immune defenses (37–40). They also regulate CD4^+^T cells development, proliferation, and differentiation during viral and parasitic infections (41, 42).

Previous laboratory studies established both hydatid infection and r*Eg*.P29 immunization models, and next-generation sequencing identified miR-378a-5p as significantly upregulated. To further investigate this, target gene prediction tools such as TargetScan, miRDB, and miRWALK suggested that miR-378a-5p may regulate BRAF. Further exploration using the online STRING platform revealed that BRAF is an upstream molecule in the MAPK/ERK pathway. Based on these findings, this study explores the regulation of the miR-378a-5p/BRAF/MAPK-ERK axis in Th1 differentiation of naïve CD4^+^T cells in response to r*Eg*.P29-induced immune reactions and identifies miR-378a-5p as a potential biomarker and immunomodulatory target for CE.

## 2 Materials and methods

### 2.1 Animals

Female BALB/c mice measuring 18–25 g at 6–8 weeks of age were acquired from Ningxia Medical University’s Experimental Animal Centre. The mice were kept at 22°C in a specialized pathogen-free (SPF) environment. All experimental protocols, including euthanasia (Sodium pentobarbital (≥100 ug/kg IP) combo followed by exsanguination), were strictly carried out in accordance with the Ningxia Medical University’s Animal Welfare Guidelines.

### 2.2 Expression, purification, and endotoxin removal of r*Eg*.P29

Our laboratory was able to effectively synthesize and recombine the *Eg*.P29 gene from *Echinococcus granulosus* obtained from *Escherichia coli* (16). The recombinant strain harboring the *Eg*.P29 gene was initially streaked onto LB agar plates supplemented with kanamycin and incubated overnight at 37°C to obtain single colonies. A single colony was inoculated into kanamycin-containing LB broth and cultured at 37°C with shaking at 200 rpm for approximately 12 h until visible turbidity was observed. The culture was scaled up until the bacterial cells reached the logarithmic growth phase, as indicated by an OD600 value of 0.6, at which point isopropyl β-D-1-thiogalactopyranoside (IPTG) was added to induce *Eg*.P29 expression. Following induction, the cells were harvested by centrifugation, treated with lysozyme and phenylmethylsulfonyl fluoride (PMSF), and lysed by sonication. A pre-equilibrated resin suspension was combined with the supernatant and incubated overnight at 4 °C under mild agitation. The recombinant *Eg*.P29 protein was purified using affinity chromatography. Subsequently, endotoxins were eliminated from purified protein and used in animal immunization experiments.

### 2.3 Establishment of a mouse model immunization of r*Eg*.P29

In the r*Eg*.P29 vaccination experiment, 21 female BALB/c mice (6–8 weeks old) were randomly assigned to three groups: phosphate-buffered saline (PBS), Freund’s complete adjuvant (FCA), and r*Eg*.P29+FCA. The mice in the PBS group received 100 uL of PBS, the FCA group received 20 μg of FCA diluted with 100 uL of PBS, and the r*Eg*.P29+FCA group received 20 μg r*Eg*.P29 and 20 μg of FCA in 100 uL of PBS. To minimize protein degradation during emulsification, the adjuvant and PBS were pre-emulsified briefly before adding the proteins, and the protocol was conducted on ice. The emulsified mixture was then loaded into a 1 mL syringe for immunization. The immunization protocol consisted of two stages, primary and booster immunizations. For booster immunization, Freund’s incomplete adjuvant (FIA) was used instead of FCA. Both primary and booster immunizations were administered via a subcutaneous three-point injection.

### 2.4 Isolation of CD4^+^T cells and naive CD4^+^T cells

Mice were euthanized by cervical dislocation and soaked in 75% alcohol. Sterile spleens were then obtained by removing the epidermis. Lymphocytes were isolated from the spleen using a mouse spleen lymphocyte separation medium kit (Tianjin HaoYang Biological Manufacture Co., Ltd., China). Subsequently, CD4^+^T cells were purified from the lymphocytes using a mouse Cells isolation kit (Miltenyi Biotec, Inc., Cologne, Germany), briefly, lymphocytes (1×10^7^ cells) were labeled with Biotin-Antibody Cocktail in 40 μL MACS buffer (4°C, 5 min), followed by incubation with Anti-Biotin MicroBeads (20 μL in 20 μL buffer; 4°C, 10 min). Cells were separated through pre-wetted MS columns mounted on a magnetic stand over ice. After centrifugation (350 × g, 10 min, 4°C), purified cells were resuspended in complete RPMI-1640 medium for subsequent experiments. Naive CD4^+^T cells were further purified using a mouse naive CD4^+^T cells isolation kit (Miltenyi Biotec, Inc., Cologne, Germany), specifically, lymphocytes (1×10^7^ cells) were first labeled with Naive CD4^+^ T Cell Biotin-Antibody Cocktail in 40 μL MACS buffer (4°C, 5 min). Subsequently, 20 μL Anti-Biotin MicroBeads and 10 μL CD44 MicroBeads were added in 20 μL buffer (4°C, 12 min). Cells were separated through ice-cooled MS columns pre-rinsed with MACS buffer, with sequential loading of cell suspension interrupted by buffer washes. Target cells were collected by centrifugation (350 × g, 10 min, 4°C) and resuspended in complete RPMI-1640 medium”.

### 2.5 Differentiation of naive CD4^+^T cells

The naive CD4^+^T cells were cultured in 24-well plates with 5 × 10^5^ cells, (precoated with anti-CD3, 1 μg/ml, Thermo Fisher Scientific Co., Ltd, Xian,USA) and were then transfected with miR-378a-5p mimics (60 nM, Shanghai GenePharma Co.,Ltd) and inhibitor (150 nM, QIAGEN, Shanghai GenePharma Co.,Ltd) using HiPerFect transfection reagent (QIAGEN, USA). After 6 h, soluble anti-CD28 (0.2 μg/ml, Thermo Fisher Scientific Co., Ltd, USA) was added along with cytokines that differentiate naive CD4^+^T cells into different T-cell subtypes. For Th1 cells, IL-2 (20 ng/ml, BioLegend, Inc, California, USA.), IL-12 (50 ng/ml, BioLegend, Inc, California, USA.), and anti-IL-4 antibody (10 ng/ml, BioLegend, Inc, California, USA) were added to the well plates for 48 h. To differentiate the cells into Th2 cells, IL-2 (20 ng/ml, BioLegend, Inc, California, USA), IL-4 (10 ng/ml, BioLegend, Inc, California, USA.), and anti-IL-IFN-γ antibody (10 ng/ml, BioLegend, Inc, California, USA.) were added to the well plates for 48 h.

### 2.6 Bioinformatics prediction of target genes

Three miRNA target prediction databases were used, namely miRWALK (version 3.0), miRDB (http://mirdb.org/miRDB/), and TargetScan (version 8.0).The analysis was conducted with the species parameter set to *Mus musculus* (mouse) and the miRNA sequence of interest was used as the query. The predicted target genes from each database were subsequently analyzed for common targets using the Venn diagram approach implemented in the Draw Venn Diagram tool. The overlapping target genes identified by all three prediction platforms were considered high-confidence candidates and visualized using a Venn diagram representation.

### 2.7 Dual-luciferase reporter assay

293T (Key Laboratory of Common Infectious Disease Prevention and Control in Ningxia) cells were reconstituted and cultivated in full media. Wild-type and mutant BRAF plasmids, in conjunction with miR-378a-5p mimics or negative controls (NC), were prepared for transfection. At 70% cellular confluence, transfection complexes were formulated by combining 10 μL DMEM with 0.3 μL transfection reagent and incubated at room temperature for 5 minutes. Concurrently, 10 μL of DMEM was combined with 0.1 μg of plasmid DNA (either wild-type or mutant BRAF) and miR-378a-5p mimics/negative control, followed by a 5-minute incubation at ambient temperature. The transfection reagent was amalgamated with the DNA solution, gently agitated by tapping the tube, and incubated for 10 minutes at an ambient temperature. The completed transfection complexes were uniformly placed into 293T cell culture plates and incubated in a humidified environment (37°C, 5% CO_2_) for 48 hours. After incubation, cells were lysed by the addition of 100 μL of Passive Lysis Buffer (PLB) each well in a 96-well plate. Following careful pipetting, the plates were agitated at an ambient temperature for 15 minutes. Cell lysates were aliquoted into 1.5 mL microcentrifuge tubes and subjected to centrifugation at 13,400 ×g for 8 minutes at 4 °C. Supernatants were obtained for further analysis. To quantify luciferase activity, 100 μL of Luciferase Assay Reagent II was mixed with 20 μL of cell lysate in each well. The activity of firefly luciferase was quantified as an internal reference. Subsequently, 100 μL of Stop & Glo^®^ Reagent was administered to each well for the assessment of Renilla luciferase activity. Relative luciferase activity was determined by the ratio of Renilla luciferase activity to firefly luciferase activity.

### 2.8 Prediction of interaction pathways with target gene

Log in to the STRING(https://cn.string-db.org/) online database and enter the target gene and species source to obtain the proteins or signaling pathways interacting with the target gene.

### 2.9 qRT-PCR

TRIzol (Thermo Fisher Scientific, USA) was used to extract total RNA from CD4^+^T cells and naïve CD4^+^T cells. The First Strand cDNA Synthesis Kit (Thermo Fisher Scientific, USA) was used to synthesis cDNA for mRNA analysis. An ABI 7500 Fast Real-Time PCR System (Thermo Fisher Scientific, USA) was used to conduct quantitative real-time PCR (qPCR). The Bestar^®^ Sybr Green qPCR Master Mix (DBI^®^ Bioscience) was used to assess the levels of mRNA expression. Both mRNA and miRNA were normalized using U6 and GAPDH as endogenous controls. The 2−ΔΔCT technique was used to determine the relative expression levels of genes. **Table 1** lists the primer sequences utilized in this investigation.

**Table 1:** Sequences of primers involved in qRT-PCR.

Gene	Forward (5’-3’)	Reverse (5’-3’)
miR-378a-5p	CAAACCTCCTCCTGACTCCAG	TATGCTTGTTCTCGTCTCTGTGTC
U6	CAGCACATATACTAAAATTGGAACG	ACGAATTTGCGTGTCATCC
IFN-γ	GCCACGGCACAGTCATTGA	TGCTGATGGCCTGATTGTCTT
IL-4	ATCATCGGCCATTTTGAACGAGG	TGCAGCTCCATGAGAACACTA
T-bet	CTTGGATCCTTCGCCTACCC	CTTCCCAGACACCTCCAACC
GATA-3	TCTCACTCTCGAGGCAGCATGA	GGTACCATCTCGCCGCCACAG
BRAF	CCACAGATGCATCACGGAAC	CATCTTGCGGGTACCACTGT
MEK-1	TGCCCAAGAAGAAGCCGAC	CTCGTCAAGCTCCAGCTCC
MEK-2	CCACCTGATGCCAAGGAACT	GTCCATCCCATGACCACTGA
ERK-1	CACTGGCTTTCTGACGGAGT	CCGGTTGGAGAGCATCTCAG
ERK-2	TCCAACCTCCTGCTGAACAC	CCAACGTGTGGCTACGTACT
GAPDH	GGTTGTCTCCTGCGACTTCA	TGGTCCAGGGTTTCTTACTCC

### 2.10 Western blot

Whole-cell lysis buffer (KeyGen Biotech, Jiangsu, China) was used to extract the total protein from the treated cells. A BCA protein quantification assay (KeyGen Biotech, Jiangsu, China) was used to measure the protein content. Protein samples were denatured for eight minutes at 100°C after being combined with 5X SDS-PAGE loading buffer (Shanghai Epizyme Biomedical Technology Co., Ltd). After being separated using 12% SDS-PAGE, the denatured proteins were put onto 0.2 μm PVDF membranes. For two hours at room temperature, the membranes were blocked using 5% skim milk in PBST. The membranes were incubated with primary antibodies against Phospho-B-Raf (Ser445), Phospho-MEK1/2, Phospho-p44/42(Erk1) (Tyr204)/(Erk2) (Tyr187) (D1H6G) Mouse mAb, (Cell Signaling Technology, USA)(1:1000), and β-actin, IFN-γ, IL-4 (Bioworld Biotech, Nanjing, China)(1:1000) overnight at 4°C. Following PBST washing, membranes were incubated for two hours at room temperature with secondary antibodies conjugated to horseradish peroxidase (HRP) (Cell Signaling Technology, Danvers, MA, USA)(1:10000). The ChemiDoc Touch Imaging System (Bio-Rad Laboratories, Shanghai, China) and an ECL detection kit (KeyGen Biotech, Jiangsu, China) were used to observe protein bands. To normalize the results, β-actin was employed as an internal loading control.

### 2.11 Staining and flow cytometry

To evaluate the purity of magnetically isolated naive CD4^+^ T cells, surface staining was performed using the following antibodies: FITC-anti-CD4, PE-anti-CD44, APC-anti-CD25, and PerCP-Cy5.5-anti-CD62L antibodies (all from BioLegend, San Diego, CA, USA).

### 2.12 Statistical analysis

The data was analyzed using GraphPad Prism software and given as mean ± SD. Student’s *t*-test was used to compare two groups. *P*-values < 0.05 were deemed statistically significant.

## 3 Results

### 3.1 The expression, purification, and identification of r*Eg*.P29, as well as its effects on miR-378a-5p and the BRAF-MAPK/ERK pathway in CD4^+^T cells

**Figure 1:**
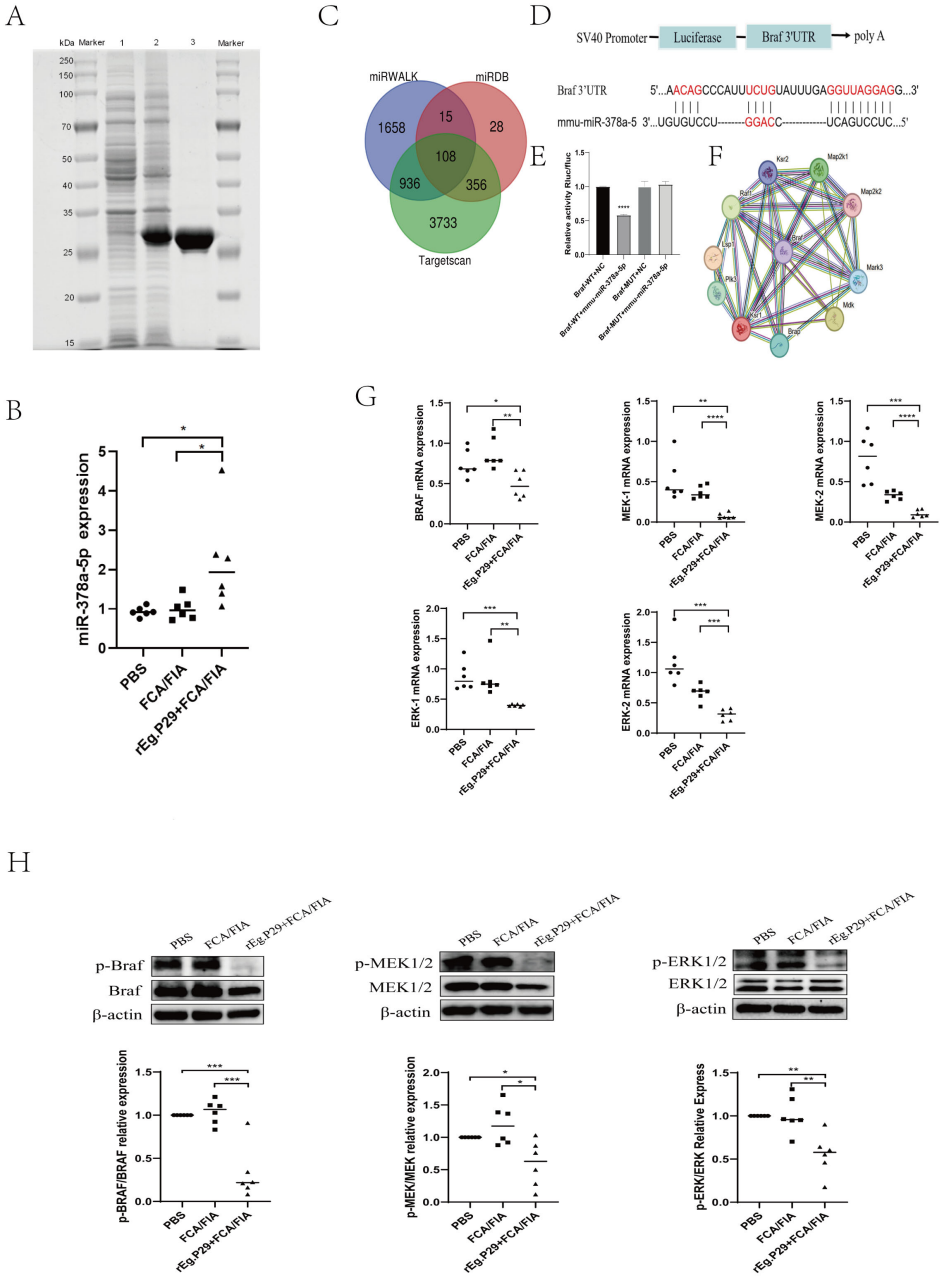
**(A)** SDS-PAGE Analysis of r*Eg*.P29 after induction, expression, and purification: line 1. Bacterial culture without IPTG induction; line 2. Bacterial culture after IPTG induction; line 3. Purified and endotoxin-free r*Eg*.P29 protein. **(B)** Expression of miR-378a-5p after r*Eg*.P29 immunization. **(C)** Venn diagram of miR-378a-5p target gene predictions from three databases. **(D, E)** The constructs used in the double luciferase assay were psiCHECK-2 vectors, the binding sites of miR-378a-5p and BRAF were illustrated, and the double luciferase reporter assay was statistically analyzed. **(F)** Protein-protein interaction network of target gene BRAF. **(G)** Effects of r*Eg*.P29 Immunization on the mRNA Expression of Key Molecules in the BRAF and MAPK/ERK Pathway in Mice. **(H)** Effects of r*Eg*.P29 Immunization on the Protein Expression of Key Molecules in the BRAF and MAPK/ERK Pathway in Mice. ****P<0.0001, ***P<0.001, **P<0.01, *P<0.05; ns, not significant.

A His-tag purification kit was used to purify the r*Eg*.P29 protein, and endotoxins were then eliminated (**Figure 1A**). The purified protein solution was sterilized by filtration through a 0.22-μm filter to meet experimental standards. Spleen lymphocytes were isolated from mice, and CD4^+^T cells. with >95% purity were obtained via magnetic-activated cell sorting (MACS). (**Supplementary Figure 1A**). Quantitative reverse transcription PCR (qRT-PCR) quantified miR-378a-5p expression in purified CD4^+^T cells. Comparative analysis revealed that r*Eg*.P29, when administered with FCA or FIA, significantly upregulated miR-378a-5p expression in mouse splenic CD4^+^T cells compared with the PBS and adjuvant-only control groups (**Figure 1B**). These results suggest that miR-378a-5p was may involved in the differentiation of CD4+T cells in mouse spleen induced by rEg.P29.

To elucidate the specific mechanism by which r*Eg*.P29-induced miR-378a-5p modulates CD4^+^T cells differentiation into Th1 cells, we used three databases (miRWalk, miRDB, and TargetScan) to predict miR-378a-5p target genes. Intersecting the target genes from these databases identified 108 overlapping genes (**Figure 1C**). Dual luciferase results showed that co-transfection of miR-378a-5p mimics in 293T cells significantly inhibited the luciferase activity of Braf wild-type 3’-UTR, validated BRAF as a target gene regulated by miR-378a-5p (**Figures 1D, E**). Using the STRING online database, we analyzed BRAF-interacting proteins and found a significant association between BRAF and the MAPK pathway (**Figure 1F**). Extensive research emphasizes BRAF’s crucial function in regulating the MAPK/ERK signaling pathway, which is critical for cell division and differentiation in many disorders (43–50). One week after using rEg.P29 immunized mice, spleen lymphocytes were isolated from mice, and CD4^+^T cells with >95% purity were obtained using magnetic bead sorting. mRNA and protein expression levels of BRAF, MEK1/2, and ERK1/2 in CD4^+^T cells were quantified by qRT-PCR and western blotting, respectively. The results demonstrated that r*Eg*.P29 combined with FCA/FIA significantly downregulated BRAF, MEK1/2, and ERK1/2 expression in mouse splenic CD4^+^T cells compared to the PBS and adjuvant groups (**Figures 1G, H**). These findings suggest that the BRAF and MAPK/ERK signaling pathways are may involved in miR-378a-5p-mediated regulation of CD4^+^T cells differentiation induced by r*Eg*.P29.

### 3.2 Effect of miR-378a-5p on the differentiation of naïve CD4^+^T Cells towards Th1 and Th2 subsets

**Figure 2:**
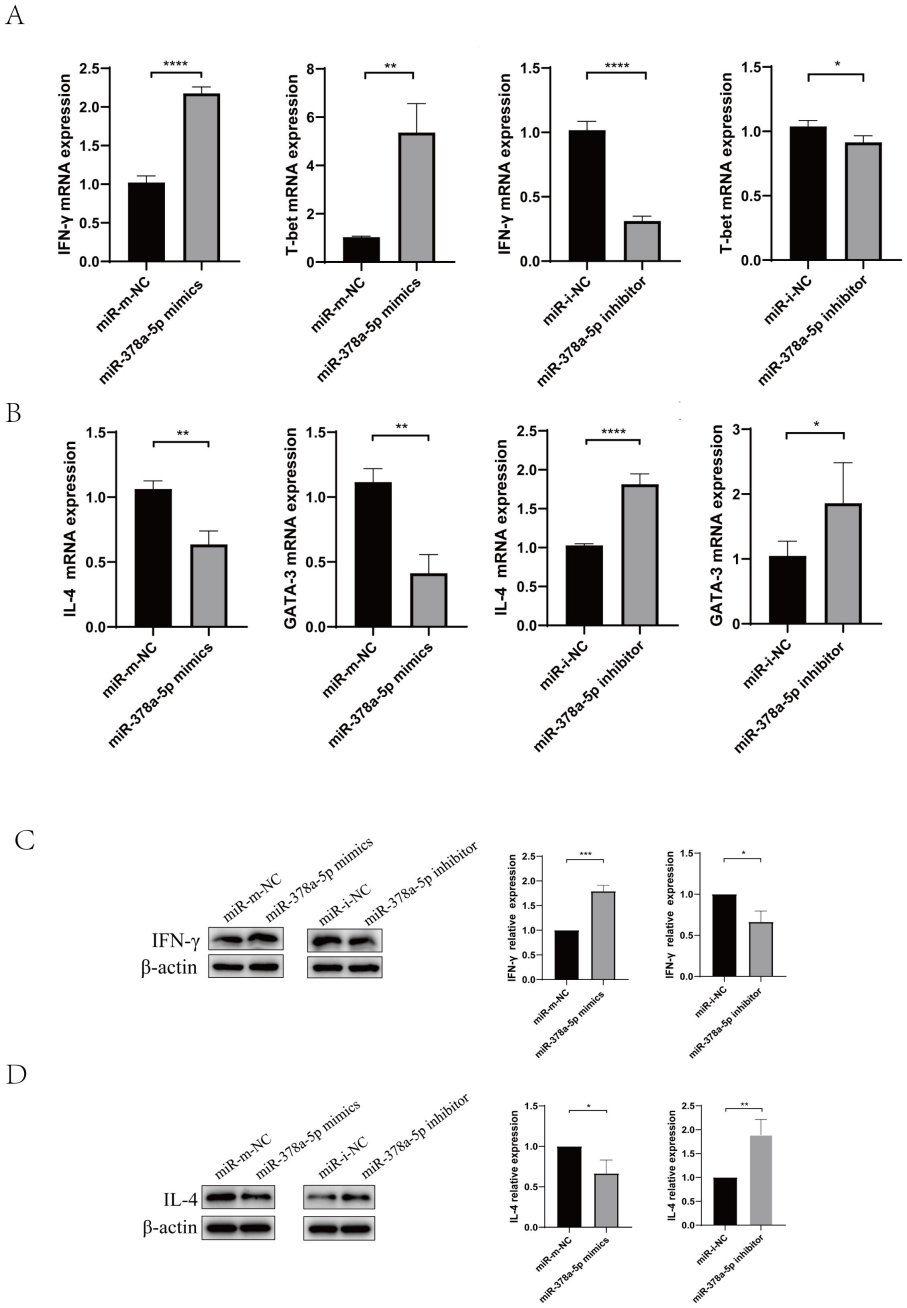
Naïve CD4^+^T cells were transfected with miR-378a-5p mimics, miR-mimics negative control (miR-mimics-NC), miR-378a-5p inhibitors, or miR-inhibitor negative control (miR-inhibitor-NC),following transfection, cells were polarized towards either the Th1 or Th2 lineage using lineage-specific cytokine cocktails. **(A)** Following Th1 polarization, mRNA expression levels of IFN-γ and T-bet were quantified using RT-qPCR. **(B)** Following Th2 polarization, mRNA expression levels of IL-4 and GATA-3 were quantified using RT-qPCR **(C)** Following Th1 polarization, protein expression levels of IFN-γ was analyzed by Western blotting. **(D)** Following Th2 polarization, protein expression levels of IL-4 was analyzed by Western blotting. ****P<0.0001, ***P<0.001, **P<0.01, *P<0.05; ns, not significant.

First, the optimal concentration of miR-378a-5p mimics or miR-378a-5p inhibitors were screened. (**Supplementary Figure 1B**). Then, Magnetic bead sorting was used to separate naïve CD4^+^T cells from mouse splenic lymphocytes, Naïve CD4^+^T cells were transfected with miR-378a-5p mimics, miR-mimics negative control (miR-mimics-NC), miR-378a-5p inhibitors, or miR-inhibitor negative control (miR-inhibitor-NC),following transfection, cells were polarized towards either the Th1 or Th2 lineage using lineage-specific cytokine cocktails. After 48 hours of stimulation, total RNA and total protein were extracted for downstream analyses. We found that miR-378a-5p mimics substantially increased Th1-associated cytokine IFN-γ and transcription factor T-bet expression compared to the control group, according to qRT-PCR analysis. In contrast, miR-378a-5p inhibitors drastically decreased these indicators. In contrast, miR-378a-5p mimics decreased the production of the Th2-associated cytokine IL-4 and transcription factor GATA-3, whereas miR-378a-5p inhibitors boosted their levels. (**Figures 2A, B**). Western blot analyses confirmed these findings, showing that miR-378a-5p mimics increased IFN-γ levels and decreased IL-4 levels, whereas miR-378a-5p inhibition had the opposite effects (**Figures 2C, D**).

### 3.3 Evaluation of miR-378a-5p on BRAF and the MAPK/ERK pathway

**Figure 3:**
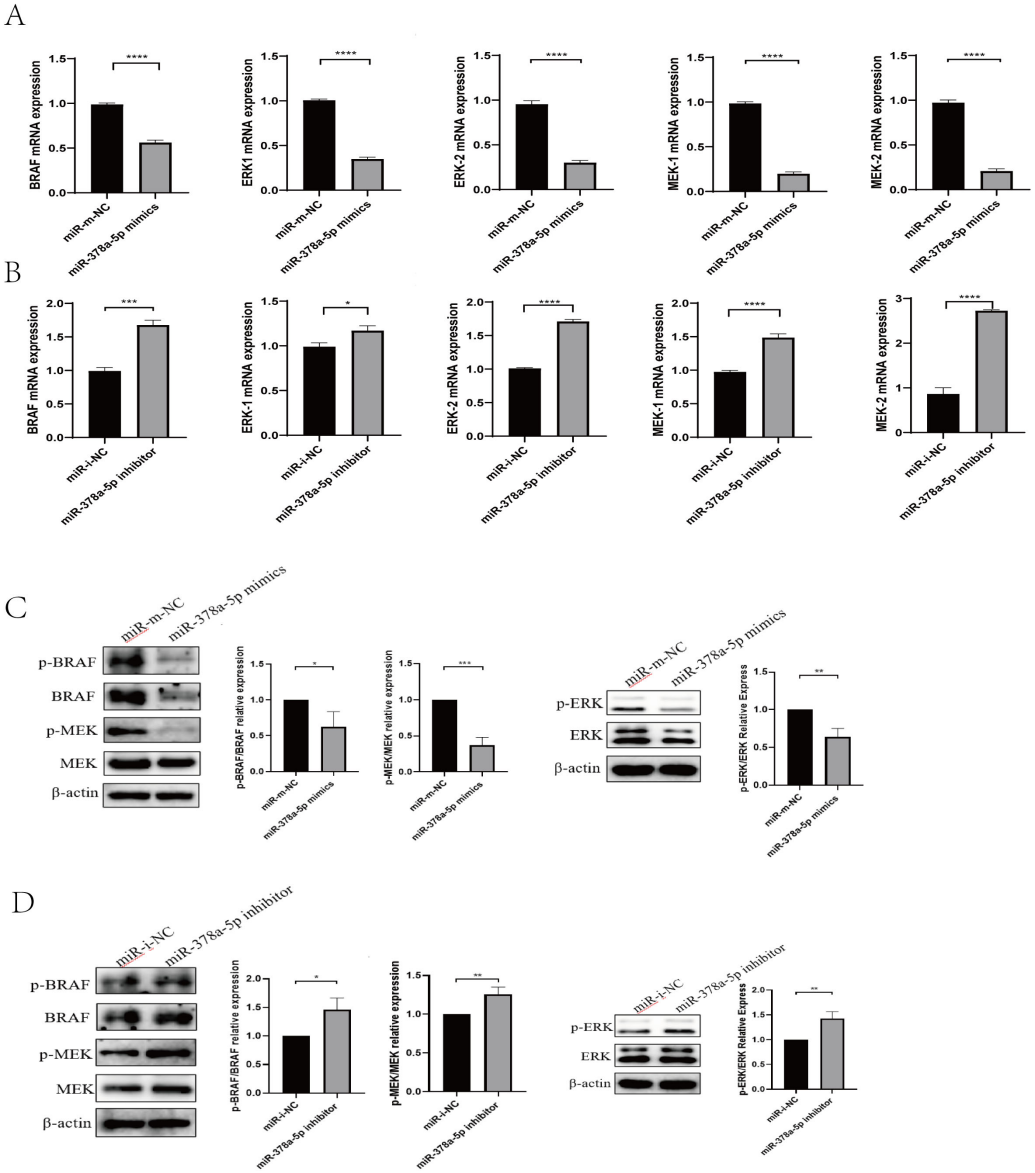
**(A)** qRT-PCR was used to analyze BRAF, MEK1/2, and ERK1/2 levels in naïve CD4^+^T cells transfected with miR-378a-5p mimics and miR- m-NC (miR- mimics-NC). **(B)** qRT-PCR was used to analyze BRAF, MEK1/2, and ERK1/2 levels in naïve CD4^+^T cells transfected with miR-378a-5p inhibitor and miR- i-NC (miR- inhibitor-NC). **(C)** Western blot was used to analyze BRAF, MEK1/2, and ERK1/2 levels in naïve CD4^+^T cells transfected with miR-378a-5p mimics and miR- m-NC (miR- mimics-NC). **(D)** Western blot was used to analyze BRAF, MEK1/2, and ERK1/2 levels in naïve CD4^+^T cells transfected with miR-378a-5p inhibitor and miR- i-NC (miR- inhibitor-NC). ****P<0.0001, ***P<0.001, **P<0.01, *P<0.05; ns, not significant.

To determine the mechanism by which r*Eg*.P29 induces miR-378a-5p to regulate CD4^+^T cells differentiation toward the Th1 phenotype, we examined the expression of key molecules in the BRAF and MAPK/ERK pathways at the mRNA and protein levels using qRT-PCR and Western blot analyses. The results demonstrated that overexpression of miR-378a-5p significantly decreased the mRNA and protein expression levels of BRAF, MEK1/2, and ERK1/2. Conversely, miR-378a-5p inhibition significantly increased the expression of these molecules (**Figures 3A–D**). These findings suggest that under the induction of rEg.P29, miR-378a-5p targeted to BRAF regulation and initiated the differentiation of CD4^+^T cells in mouse spleen to Th1 direction, and MAPK/ERK pathway may be involved in this process.

### 3.4 Effect of BRAF on differentiation of naïve CD4^+^T cells towards Th1 and Th2

**Figure 4:**
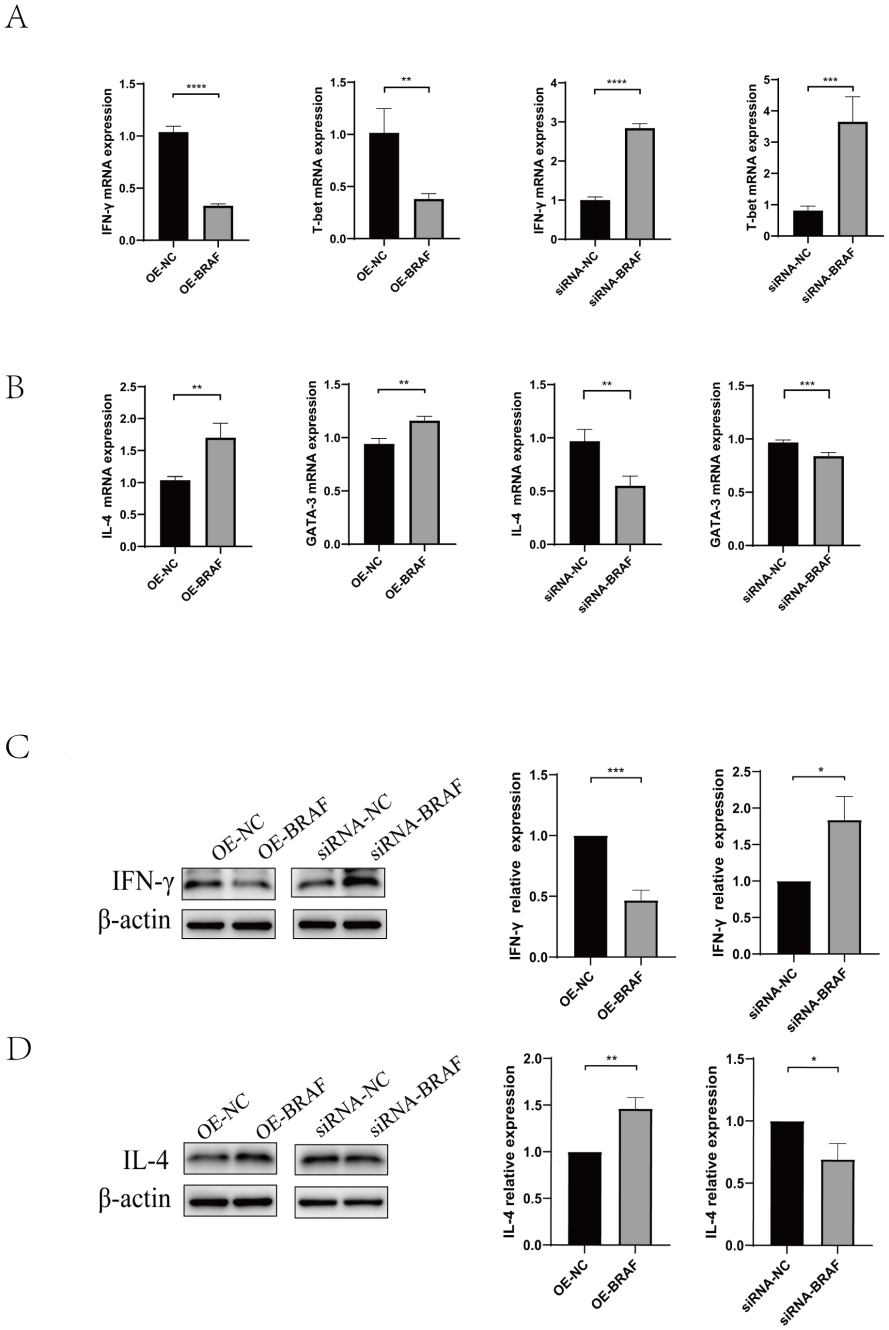
**(A)** qRT-PCR was used to analyze IFN-γ and T-bet levels in naïve CD4^+^T cells transfected with OE-*BRAF* or siRNA-*BRAF*. **(B)** qRT-PCR was used to analyze IL-4 and GATA-3 levels in naïve CD4^+^T cells transfected with OE-*BRAF* or siRNA-*BRAF* **(C)** Western blot was used to analyze IFN-γ levels in naïve CD4^+^T cells transfected with OE-*BRAF* or siRNA-*BRAF*. **(D)** Western blot was used to analyze IL-4 levels in naïve CD4^+^T cells transfected with OE-*BRAF* or siRNA-*BRAF*. ****P<0.0001, ***P<0.001, **P<0.01, *P<0.05; ns, not significant.

Magnetic bead-based cell sorting was used to extract naïve CD4^+^T cells from mouse splenic lymphocytes. To elucidate the regulatory function of BRAF in CD4^+^T cells differentiation, we first Screened of OE-*BRAF* (overexpression - *BRAF*) via Recombinant Lentivirus Packaging (**Supplementary Figure 2A**),.and synthesized siRNA-*BRAF* (siRNA-induced *BRAF* silencing) by GenePharma company, then, determined the optimal transfection about OE-*BRAF* and siRNA-*BRAF.* we performed qRT-PCR to assess the effects of OE-*BRAF* and siRNA-*BRAF*. Our findings revealed that OE-*BRAF* significantly suppressed the expression of Th1-associated cytokine IFN-γ and its key transcription factor T-bet relative to control groups. In contrast, siRNA-*BRAF* significantly enhanced the expression of Th1 markers (**Figures 4A**). Conversely, OE-*BRAF* promoted the expression of the Th2-associated cytokine IL-4 and its transcription factor GATA-3, whereas siRNA-*BRAF* significantly attenuated their expression (**Figures 4B**). These observations were further corroborated by Western blot analysis, which demonstrated that OE-*BRAF* reduced IFN-γ production while augmenting IL-4 expression (**Figures 4C, D**). Taken together, these data indicate that OE-*BRAF* inhibits Th1 differentiation in primary CD4^+^T cells, whereas siRNA-*BRAF* promote Th1 differentiation.

### 3.5 Impact of co-transfection with miR-378a-5p and *BRAF* on Th1 and Th2 differentiation

**Figure 5:**
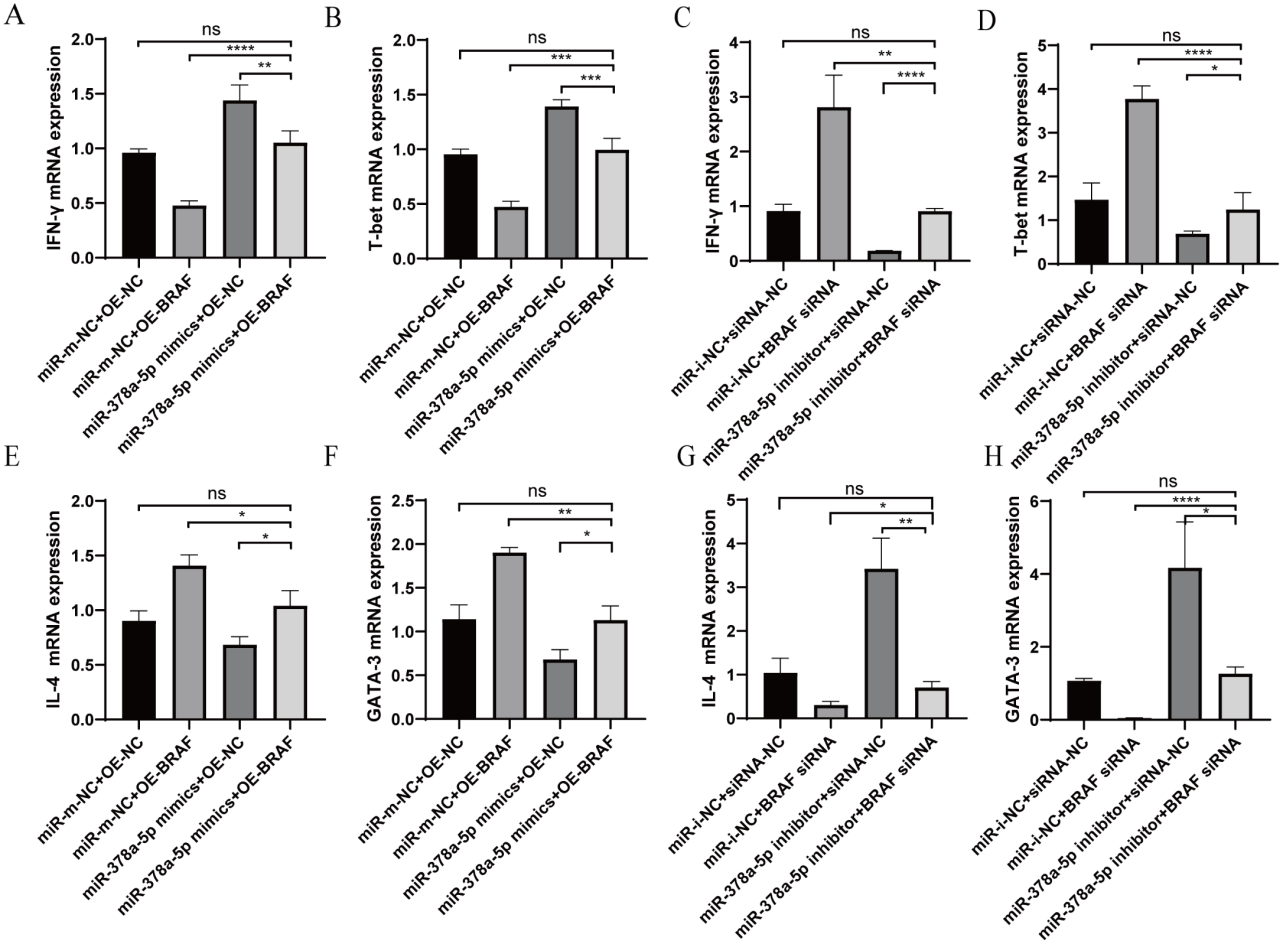
**(A–D)** qRT-PCR was used to analyze IFN-γ and T-bet levels in naïve CD4^+^T cells after Co-Transfection with miR-378a-5p and *BRAF*. **(E–H)** qRT-PCR was used to analyze IL-4 and GATA-3 levels in naïve CD4^+^T cells after Co-Transfection with miR-378a-5p and *BRAF*. ****P<0.0001, ***P<0.001, **P<0.01, *P<0.05; ns, not significant.

Using qRT-PCR, the impact of co-transfection *BRAF* and miR-378a-5p on Th1/Th2 differentiation was evaluated. Th1-associated cytokine IFN-γ and transcription factor T-bet levels in CD4^+^T cells co-transfected with OE-*BRAF* and miR-378a-5p mimics were greater than those in cells transfected with OE-*BRAF* alone, but considerably lower than those in cells transfected with miR-378a-5p mimics alone. Contrastingly, when cells were co-transfected with the miR-378a-5p inhibitor and siRNA-*BRAF*, Th1 cytokineTh1-associated cytokine IFN-γ and transcription factor T-bet levels significantly decreased in comparison to cells transfected with siRNA-*BRAF* alone, but they did significantly rise in comparison to cells transfected with the miR-378a-5p inhibitor alone. (**Figures 5A–D**). Moreover, Th2-associated cytokine IL-4 and transcription factor GATA-3 levels were considerably increased by co-transfection with miR-378a-5p mimics and OE-*BRAF* in comparison to cells transfected with miR-378a-5p mimics alone but decreased in comparison to cells transfected with OE-*BRAF* alone. Conversely, Th2-associated cytokine IL-4 and transcription factor GATA-3 levels were significantly lower after co-transfection with the miR-378a-5p inhibitor and siRNA-*BRAF*, than after cells transfected with the miR-378a-5p inhibitor alone, but higher after co-transfection with siRNA-*BRAF* (**Figures 5E–H**).

## 4 Discussion

Recent research has shown that vaccinating intermediate hosts is a viable technique for preventing the spread of CE. Vaccines such as *Eg*95, *Eg*29, and *Eg*N123 have shown promising immune protection (51–53). However, their high costs hinder their widespread use. Therefore, investigating immunological mechanisms is essential to guide the development of more effective vaccines.

CD4^+^T cells are crucial to immunological responses, and their differentiation is directly influences by *E. granulosus* infection. Th1 immune differentiation is associated with immunological protection, whereas Th2 immunity is linked to parasite growth in later stages of infection (24, 54).

Previous investigations have indicated that r*Eg*.P29 provides strong immunological protection in both sheep and mice. Additionally, miRNA microarray analysis of immunized and infected mice suggested upregulation of miR-378a-5p. Although research on the role of the miR-378 family in T cell immune regulation is limited, most studies focus on its involvement in cancer. The miR-378 family is highly expressed in normal tissues and exerts protective effects, including guarding the heart from ischemic injury, inhibiting cardiomyocyte hypertrophy, and preventing liver fibrosis (55). Mice lacking miR-378 or with a conditional knockout, exhibit impaired maintenance of muscle mass, reduced exercise capacity, autophagy dysfunction, mitochondrial accumulation, and severe apoptosis in skeletal muscle cells. In contrast, miR-378 overexpression promotes autophagy and prevents skeletal muscle cell death (56–60). In light of these findings, we examined miR-378a-5p expression to explore its potential role in r*Eg*.P29-induced immune regulation. Our findings demonstrated that in naïve CD4^+^T cells, miR-378a-5p inhibited Th2 development while promoting Th1 differentiation.

To further understand the molecular mechanism behind miR-378a-5p-mediated CD4^+^T cells differentiation, we utilized miRNA target prediction algorithms, bioinformatics analysis, and literature review to identify BRAF as a potential target. A dual-luciferase reporter gene assay confirmed that BRAF is a direct target of miR-378a-5p. Functional studies showed that miR-378a-5p overexpression significantly reduced BRAF mRNA levels and protein phosphorylation, whereas miR-378a-5p inhibition had the opposite effect. To further investigate this, we generated lentiviral constructs for BRAF overexpression and RNA interference, which were transfected into naïve CD4^+^T cells from mouse spleens. These experiments confirmed that miR-378a-5p promotes Th1 differentiation by inhibiting BRAF. Using the STRING database for protein interaction analysis, we found that BRAF is closely linked to the MAPK/ERK signaling pathway. ERK activation in T-cell precursors promotes CD4^+^T cells differentiation (61), and ERK-mediated regulation of the IL-4 receptor (IL-4R) influences Th2 differentiation by increasing IL-10 production (62, 63). Our data demonstrated that miR-378a-5p overexpression significantly reduced the mRNA levels and phosphorylation of MEK1/2 and ERK1/2, while miR-378a-5p inhibition produced the opposite effect. These results suggest that miR-378a-5p-mediated Th1 differentiation via BRAF inhibition may involve the MAPK/ERK pathway.

In conclusion, our *in vivo* and *in vitro* experiments demonstrated that r*Eg*.P29 induction leads to miR-378a-5p targeting BRAF, which regulates the differentiation of naïve CD4^+^T cells from mouse spleens toward Th1 cells, potentially via the MAPK/ERK pathway. This study offers new insights and a theoretical foundation for the development of anti-*Echinococcosis* vaccines.

## Data Availability

The original contributions presented in the study are included in the article/**Supplementary Material**. Further inquiries can be directed to the corresponding author.

## Supplementary material

Supplementary Figure 1
**(A)** Purity evaluation of first CD4^+^T lymphocytes sorted using magnetic beads. **(B)** Evaluation of various transfection concentrations of miR-378a-5p mimics and inhibitors.

Supplementary Figure 2
**(A)** Lentivirus Packaging in 293T cells. OE-NC: Fluorescence image of cells transduced with the empty lentiviral vector showing baseline fluorescence; OE-BRAF: Fluorescence image of cells transduced with the BRAF overexpression lentiviral vector (OE-BRAF) showing enhanced fluorescence. **(B)** BRAF Overexpression Lentivirus: the effect of BRAF overexpression lentivirus on BRAF expression. **(C)** BRAF Interference Fragments: The impact of different BRAF interference fragments on BRAF expression.
