# Supplementary figures and images for: miR-378a-5p targeting BRAF regulates CD4+T cells differentiation to Th1 under rEg.P29 induction
Source: Front Immunol. 2025 Sep 4;16:1620225. doi: 10.3389/fimmu.2025.1620225 (PMC12443751; doi:10.3389/fimmu.2025.1620225)

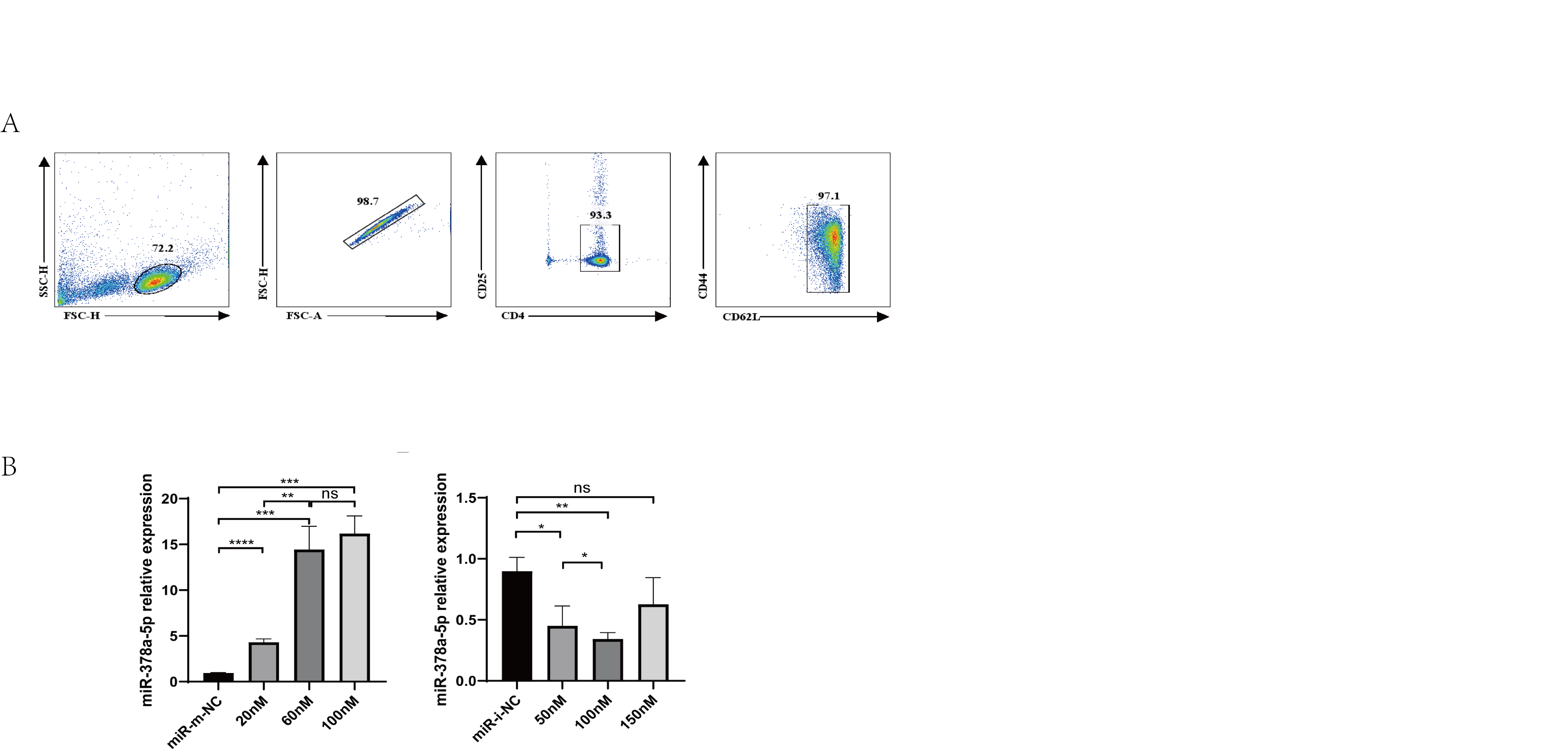

Supplement: Supplementary Figure 1 — (A) Purity evaluation of first CD4+T lymphocytes sorted using magnetic beads. (B) Evaluation of various transfection concentrations of miR-378a-5p mimics and inhibitors. [file Image1.tif]

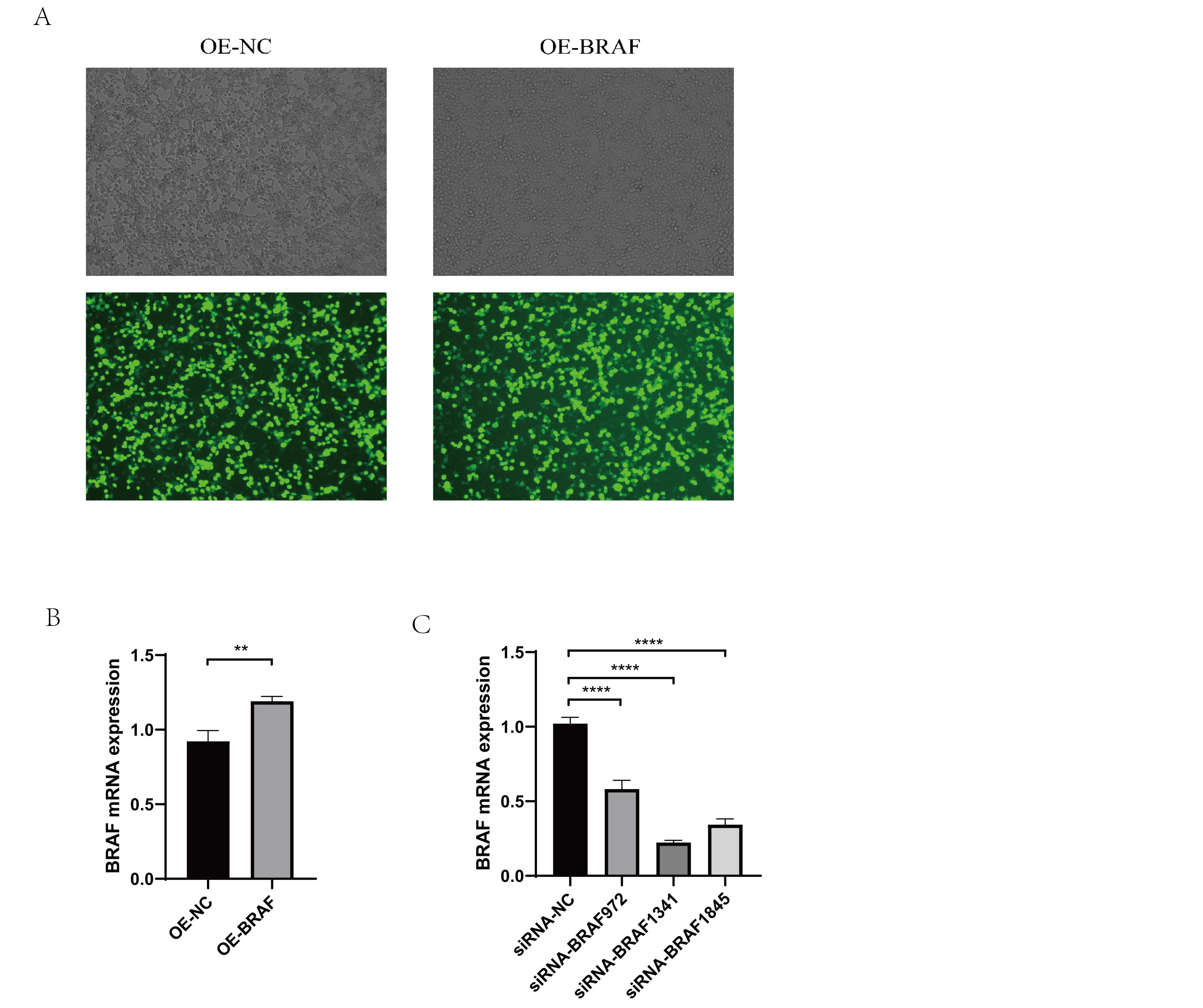

Supplement: Supplementary Figure 2 — (A) Lentivirus Packaging in 293T cells. OE-NC: Fluorescence image of cells transduced with the empty lentiviral vector showing baseline fluorescence; OE-BRAF: Fluorescence image of cells transduced with the BRAF overexpression lentiviral vector (OE-BRAF) showing enhanced fluorescence. (B) BRAF Overexpression Lentivirus: the effect of BRAF overexpression lentivirus on BRAF expression. (C) BRAF Interference Fragments: The impact of different BRAF interference fragments on BRAF expression. [file Image2.jpeg]
